# Account of Diseases in an Eastern District of London, from September 20, to October 20, 1803

**Published:** 1803-11-01

**Authors:** 


### *Account of Diseases in an Eastern District of London, from September 20, to October 20, 1803.*

ACUTE DISEASES.

| | |
|---|---|
| Typhus | 10 |
| Cholera | 14 |
| Dysenteria | 9 |
| Pneumonia | 2 |
| Angina | 2 |
| Variola | 1 |

CHRONIC DISEASES.

| | |
|---|---|
| Tussis | 15 |
| Dyspnœa | 9 |
| Tussis cum Dyspnœa | 9 |
| Hæmoptysis | 2 |
| Hepatitis Chronica | 1 |
| Enterodynia | 7 |
| Diarrhœa | 15 |
| Hæmorrhois | 3 |
| Gastrodynia | 8 |
| Anasarca | 4 |
| Amenorrhœa | 9 |
| Leucorrhœa | 11 |
| Menorrhagia | 4 |
| Dyspepsia | 10 |
| Rheumatismus Chronicus | 12 |

PUERPERAL DISEASES.

| | |
|---|---|
| Enteritis | 4 |
| Ephemera | 7 |
| Menorrhagia Lochialis | 5 |
| Mastodynia | 6 |

INFANTILE DISEASES.

| | |
|---|---|
| Aphthæ | 4 |
| Convulsio | 2 |
| Vermes | 4 |
| Diarrhœa | 7 |

The present state of disease is very similar to that which was taken notice of in our last Report. As we advance farther into the autumnal season, we may expect that the number of instances of those complaints which are peculiar to that period will increase. Intestinal affections prevail to a considerable degree. Diarrhœa still occurs very frequently, and connects itself very much with other complaints. Cholera and Dysenteria are now beginning to form a large proportion of the catalogue of diseases. Of those referred to in the preceding list, not any one terminated fatally.

Fevers of the typhus kind are becoming very frequent, and the symptoms attending them have, in several instances, been severe and threatening. In one case, which terminated fatally, the internal fauces exhibited a very dark appearance, which spread also over the lips and the lower part of the nose. A florid eruption on the nose or lips, attended with soreness, or small fissures at the tip or on the side of the tongue, sometimes appear in fevers of this kind, and are followed by a remission of symptoms; but, in the instance referred to, a dark coloured and gangrenous appearance of the sore about the nose, lips, and chin, conspired with other symptoms to form an unfavourable prognosis, and in a few days the patient died.

